# Association of HIV Preexposure Prophylaxis Use With HIV Incidence Among Men Who Have Sex With Men in China: A Nonrandomized Controlled Trial

**DOI:** 10.1001/jamanetworkopen.2021.48782

**Published:** 2022-02-16

**Authors:** Hongyi Wang, Zixin Wang, Xiaojie Huang, Yaokai Chen, Hui Wang, Sitong Cui, Jing Zhang, Zhenxing Chu, Qinghai Hu, Haibo Ding, Hanzhu Qian, Huachun Zou, Weiming Tang, Yangyang Gao, Xia Jin, Zhaozhen Liu, Lukun Zhang, Jin Zhao, Xiaoqing He, Yongjun Jiang, Wenqing Geng, Junjie Xu, Hong Shang

**Affiliations:** 1 NHC Key Laboratory of AIDS Immunology (China Medical University), National Clinical Research Center for Laboratory Medicine, The First Affiliated Hospital of China Medical University, Shenyang; 2 Key Laboratory of AIDS Immunology, Chinese Academy of Medical Sciences, Shenyang; 3 Key Laboratory of AIDS Immunology of Liaoning Province, Shenyang, China; 4 Collaborative Innovation Center for Diagnosis and Treatment of Infectious Diseases, Hangzhou, China; 5 JC School of Public Health and Primary Care, Faculty of Medicine, Chinese University of Hong Kong, Hong Kong SAR, China; 6 Center for Infectious Diseases, Beijing Youan Hospital, Capital Medical University, Beijing, China; 7 Chongqing Public Health Medical Center, Chongqing, China; 8 Department of Infectious Diseases, National Clinical Center for Infectious Diseases, Third People’s Hospital of Shenzhen (Second Affiliated Hospital of Southern University of Science and Technology), Shenzhen, China; 9 School of Public Health, Yale University, New Haven, Connecticut; 10 School of Public Health (Shenzhen), Sun Yat-sen University, Shenzhen, China; 11 University of North Carolina at Chapel Hill Project–China, Guangzhou, China; 12 Shenzhen Center for Disease Control and Prevention Shenzhen, Guangdong, China

## Abstract

**Importance:**

Evidence on HIV preexposure prophylaxis (PrEP) among Chinese men who have sex with men (MSM) is critical to guide its large-scale implementation in low- and middle-income countries.

**Objective:**

To evaluate incident HIV infection, adherence, safety, and changes in sexual behaviors among MSM using daily PrEP (D-PrEP) and event-driven PrEP (ED-PrEP) in 4 cities in China.

**Design, Setting, and Participants:**

This nonrandomized controlled trial was conducted among HIV-seronegative MSM from December 11, 2018, to November 30, 2020, in Beijing, Shenyang, Chongqing, and Shenzhen. Participants self-chose D-PrEP or ED-PrEP regimens at baseline and could switch regimens during the 12-month study period. HIV-negative MSM who declined to initiate PrEP (nonusers) in the same cities joined a separate parallel prospective cohort and served as control individuals.

**Interventions:**

PrEP consisted of coformulated tenofovir disoproxil fumarate, 300 mg, and emtricitabine, 200 mg.

**Main Outcomes and Measures:**

The main outcome was incident HIV infection. Poisson regression was used to obtain the HIV incidence rate ratio (IRR).

**Results:**

A total of 1530 MSM were included in the analysis (median age, 30 [IQR, 25-37] years). At baseline, 520 MSM chose D-PrEP (median age, 29 [IQR, 25-35] years) and 503 chose ED-PrEP (median age, 29 [IQR, 25-36] years). The median HIV Risk Index score was 18 (IQR, 12-22) among D-PrEP users and 18 (IQR, 11-22) among ED-PrEP users. Among 507 PrEP nonusers, the median age was 33 (IQR, 27-43) years, and the median HIV Risk Index score was 12 (IQR, 7-18). Although PrEP users had more baseline behaviors associated with HIV risk, the HIV incidence was lower among all PrEP users (adjusted IRR, 0.09 [95% CI, 0.04-0.21]), ED-PrEP users (adjusted IRR, 0.05 [95% CI, 0.01-0.22]), and D-PrEP users (adjusted IRR, 0.12 [95% CI, 0.04-0.33]) compared with PrEP nonusers. There was no difference in HIV incidence between D-PrEP users and ED-PrEP users (IRR, 0.33 [95% CI, 0.06-2.04]). Event-driven PrEP users consumed 40% fewer tablets than D-PrEP users during the study period. Adherence, defined as the proportion of self-reported days with sexual intercourse in which PrEP was taken according to prescription of at least 90%, increased over time among ED-PrEP users (from 57.4% to 77.8%; *P* < .001 for trend) and decreased over time among D-PrEP users (from 75.1% to 72.1%; *P* = .02 for trend). Daily PrEP users reported fewer adverse events than ED-PrEP users (193 of 520 [37.1%] vs 241 of 503 [47.9%]).

**Conclusions and Relevance:**

The findings of this study suggest that D-PrEP and ED-PrEP regimens are associated with lower incidence of HIV and a good safety profile among high-risk MSM in China.

**Trial Registration:**

Chinese Clinical Trial Registry number: ChiCTR-IIN-17013762

## Key Points

**Question:**

What is the association of daily (D-PrEP) and event-driven preexposure prophylaxis (ED-PrEP) with HIV incidence among HIV-seronegative men who have sex with men in resource-limited real-world settings?

**Findings:**

In this nonrandomized controlled trial of 1530 participants, HIV incidence was significantly lower among all PrEP users, D-PrEP users, and ED-PrEP users compared with PrEP nonusers. PrEP users reported no serious adverse events; ED-PrEP users consumed 40% fewer tablets than D-PrEP users, and their medication adherence increased over time.

**Meaning:**

These findings suggest that D-PrEP and ED-PrEP regimens are both associated with lower HIV incidence and a good safety profile among men who have sex with men and who are at high risk of HIV in resource-limited settings.

## Teaser

This nonrandomized controlled trial evaluates the association of daily and event-driven HIV preexposure prophylaxis with HIV incidence in a cohort of Chinese men who have sex with men and are at high risk for HIV infection.

## Introduction

HIV disproportionately affects men who have sex with men (MSM). Globally, approximately 45% of new adult HIV infections were among MSM outside sub-Saharan Africa in 2020.^1^ Pre-exposure prophylaxis (PrEP) is an evidence-based risk reduction measure that involves the initiation of combined tenofovir disoproxil fumarate and emtricitabine before and during HIV exposure in HIV-seronegative individuals to prevent HIV acquisition.^2^ Randomized clinical trials showed that both daily PrEP (D-PrEP) and event-driven PrEP (ED-PrEP) could significantly reduce the risk of HIV acquisition among at-risk groups, including MSM.^2,3,4,5^ The World Health Organization strongly recommends D-PrEP for all populations at substantial risk of HIV infection.^6^ Event-driven PrEP can also be listed as an alternative to D-PrEP.^7^

Few countries include ED-PrEP in their PrEP guidelines or HIV prevention strategies for MSM. Lack of evidence from real-world studies, especially from low- and middle-income countries (LMICs) such as China, hindered ED-PrEP implementation. Open-label prospective cohort studies in Europe showed that D-PrEP and ED-PrEP users had similarly low HIV incidence.^8,9,10^ However, without PrEP nonusers as controls, whether the observed low HIV incidence was due to participants’ low HIV risk or PrEP effectiveness was unclear. Recently, a study from Africa^11^ compared HIV incidence among D-PrEP and ED-PrEP users with that of historical controls. To our knowledge, no study has included a parallel group of PrEP nonusers. In previous open-label studies that allowed participants to choose D-PrEP or ED-PrEP regimens,^8,9,10^ most participants preferred D-PrEP because they perceived ED-PrEP as more challenging to use. Adherence is crucial for achieving high efficacy of PrEP.^2^ Current guidelines on ED-PrEP do not have detailed instructions on adherence support.^7,12,13^ Evidence on real-world effectiveness and adherence of D-PrEP and ED-PrEP are needed to inform policy making in LMICs.

China has the second largest number of people living with HIV in Asia (1.29 million).^1^ At the time of the study, PrEP was not yet approved in mainland China, and there was no indication for PrEP (including coformulated tenofovir-emtricitabine) or relevant guidelines. Very few Chinese MSM used PrEP before the study.^14^ We conducted the China Real-World Oral Intake of PrEP (CROPrEP) project among Chinese MSM to evaluate HIV acquisition among D-PrEP and ED-PrEP users compared with a separate parallel prospective cohort of MSM nonusers. This study also compared the adherence, safety, changes in sexual behaviors, and incidence of sexually transmitted infection (STI) between D-PrEP and ED-PrEP users.

## Methods

### Study Design

The CROPrEP project was a multicenter study in 4 metropolitan cities in China (Beijing, Shenyang, Shenzhen, and Chongqing) from December 11, 2018, to November 30, 2020.^15^ Eligible MSM could choose D-PrEP or ED-PrEP based on their preference. In the same cities, HIV-negative MSM with similar risk profiles who declined to initiate PrEP (nonusers) enrolled in a separate prospective cohort and served as controls. The trial protocol is provided in Supplement 1. The protocol was approved by the institutional review board of the First Affiliated Hospital of China Medical University and was performed in accordance with the Declaration of Helsinki^16^ and relevant guidelines. Written informed consent was obtained from the participants before the start of any study procedure. The study followed the Transparent Reporting of Evaluations With Nonrandomized Designs (TREND) reporting guideline.

### Participant Recruitment and Allocation

**Figure 1.**
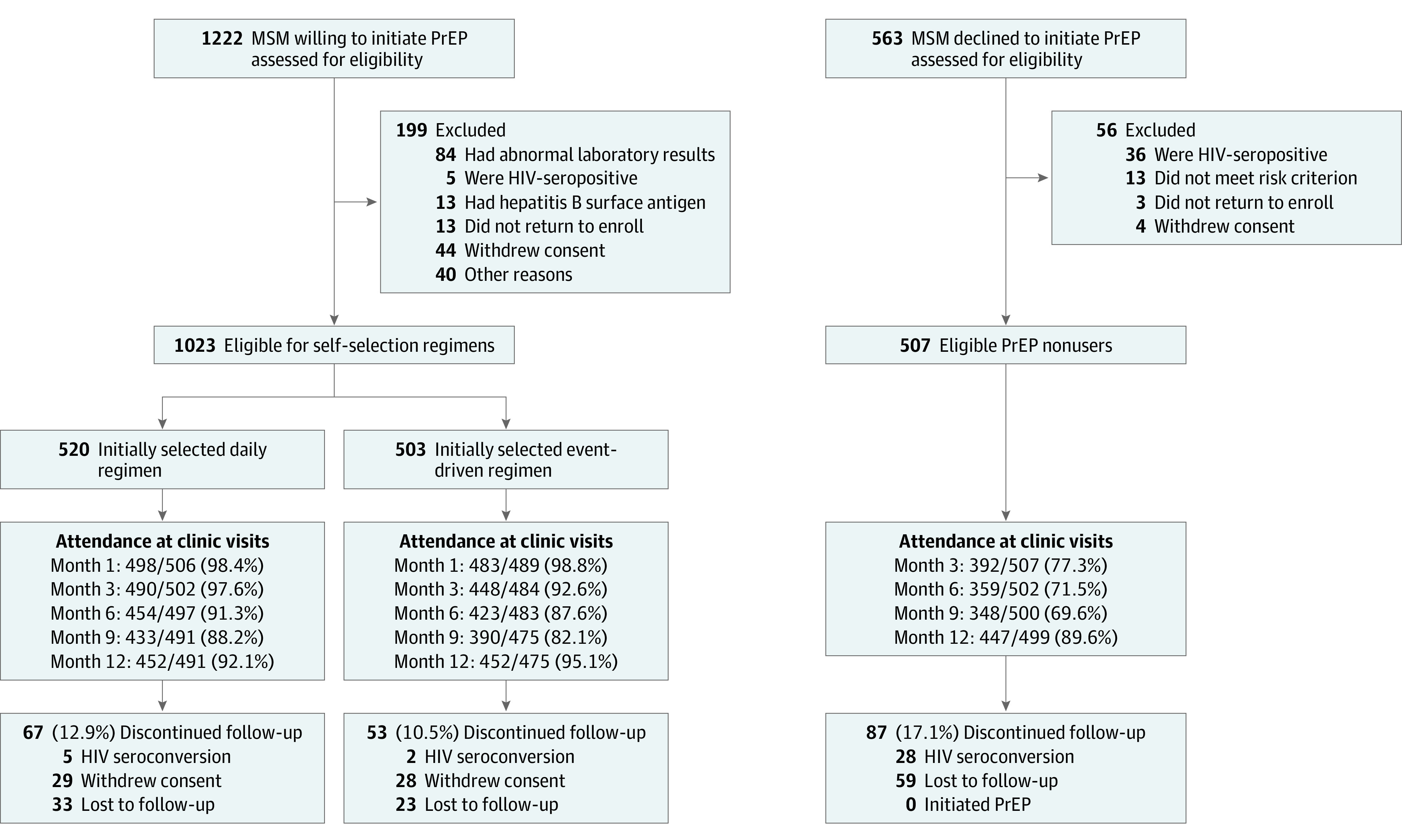
Study Profile MSM indicates men who have sex with men; PrEP, preexposure prophylaxis.

We recruited eligible participants through advertisements on social media platforms, outreach in venues for MSM, and peer referral.^15^ Participants were men aged 18 to 65 years who were HIV-seronegative, had oral and/or anal intercourse with men in their lifetime, and reported at least 1 sexual risk criterion in the past 6 months (ie, condomless receptive anal intercourse [CRAI] with men, ≥2 male sexual partners, history of STI diagnosis, or use of postexposure prophylaxis). The exclusion criteria consisted of (1) HIV infection, (2) serious chronic diseases (eg, metabolic diseases or neurological and psychiatric disorders), (3) liver and kidney dysfunction (ie, creatinine clearance [estimated glomerular filtration rate] <60 mL/min or severe liver or kidney dysfunction), (4) positive finding for hepatitis B virus surface antigen, and (5) use of antiretrovirals, interferons, or interleukins. With the exception of exclusion criteria 2 through 5, the same eligibility and exclusion criteria applied to PrEP nonusers. Nonusers were recruited using the same methods as PrEP users. From December 11, 2018, to October 31, 2019, 1222 MSM who were interested in initiating PrEP were screened for eligibility. Of these, 1023 MSM were eligible and initiated PrEP, with 520 choosing D-PrEP and 503 choosing ED-PrEP. During the same period, 507 of 563 MSM who declined to use PrEP (nonusers) enrolled in the separated cohort as control participants (Figure 1).

Eligible participants interested in initiating PrEP revisited the same clinic within 1 week to choose D-PrEP or ED-PrEP and receive a free supply of tenofovir-emtricitabine and detailed instructions. Truvada was the formulation used as PrEP in this study; each pill contains 200 mg of emtricitabine and 300 mg of tenofovir disoproxil fumarate. For D-PrEP, the regimen was 1 pill every 24 hours. For ED-PrEP, the regimen was 2 pills at 2 to 24 hours before sexual intercourse (or 1 pill if the last medication was 1-6 days ago), 1 pill every 24 hours from the first dose during the period of sexual activities, including after the last sexual intercourse, and 1 final pill approximately 24 hours later. Participants could switch PrEP regimens during the study period. If participants wanted to switch regimens, they were advised to do so after completing the next clinic visit. All PrEP users followed such advice. PrEP nonusers were followed up at the same clinic. They could initiate PrEP at any time if they wanted. However, none of them initiated PrEP during the study period.

### Study Visits and Data Collection

Daily PrEP and ED-PrEP users completed 6 study visits at the HIV clinics, including the screening at baseline and 5 follow-up visits at 1, 3, 6, 9, and 12 months. PrEP nonusers completed 4 quarterly follow-up visits for 1 year. The procedures of each clinic visit are summarized in eTable 1 in Supplement 2. PrEP users and nonusers received laboratory tests on HIV (antibodies and pooling RNA) and syphilis (*Trepeonema pallidum* particle assay and rapid plasma reagin); PrEP users underwent additional tests of liver and kidney function, blood glucose level, and bone mineral density (eTable 2 in Supplement 2). If a participant had seroconversion to HIV, he would discontinue PrEP immediately, return the remaining tablets, and undergo resistance and viral load testing and referral to HIV treatment and care. In addition, both D-PrEP and ED-PrEP users completed an identical personal weekly diary on smartphones recording weekly pill intake and sexual activities.

To maintain physical distancing during the COVID-19 pandemic (from January 1 to July 5, 2020), we dispatched PrEP by post and provided relevant supports through telehealth (ie, HIV/syphilis self-test, self-collected dried blood spots for HIV RNA and drug concentration testing, electronic questionnaire survey, and online interview). Moreover, we set up a 24-hour hotline for adverse events consultation by clinicians to ensure the safety of participants who could not visit the clinic for safety-related laboratory tests owing to the COVID-19 pandemic.

### Outcome Measures

The efficacy end point was incident HIV infection. HIV serostatus was evaluated by enhanced chemiluminescence immunoassay and confirmed with an HIV-1/2 Western blot immunoassay. Individuals with negative test results received an HIV RNA pooling nucleic acid amplification test to detect acute HIV infection.^17^ For participants with new HIV infection, we defined the HIV seroconversion point as the midpoint between the date of the last HIV-negative test result and the date of the first HIV-positive test result. Information on behaviors associated with HIV risk was also collected.

Adherence was measured by the percentage of self-reported days in which anal intercourse was covered by PrEP taken according to prescription, which was calculated as a percentage using days in which anal intercourse occurred as a denominator and the number of these days in which PrEP was correctly taken according to prescription as a numerator.^10^ Information on sexual activity and pill intake were extracted from participants’ weekly diaries. The staff cross-checked self-reported intakes with the pill dispensing records and pill counts at each follow-up clinic visit. They also documented the number of participants who switched regimens and their reasons.

Participants reported PrEP-related adverse events, and the number of participants who changed regimen or discontinued PrEP owing to adverse events was documented. Data on spinal bone mineral density, quantitative proteinuria, serum creatinine level, estimated glomerular filtration rate, and the presence of grade 3 or 4 laboratory abnormalities were obtained from the laboratory tests at each follow-up clinic visit. Two independent physicians evaluated the cause and severity of the adverse events (eTable 3 in Supplement 2). We kept track of all adverse events until the participants recovered or stabilized. Serious adverse events, including severe impairment of organ function and life-threatening events, were reported to the sponsor and the institutional review board.

### Statistical Analysis

Pairwise comparisons were conducted to identify differences in baseline characteristics between groups, including sociodemographics, behaviors associated with HIV risk, history of STI, and HIV Incidence Risk Index score for MSM (eTable 4 in Supplement 2).^18^ We used χ^2^ tests and/or 1-way analysis of variance or Wilcoxon rank sum test. Based on the actual PrEP regimen used in each follow-up period, we compared HIV and syphilis incidence, adherence, and change in sex behaviors among D-PrEP users, ED-PrEP users, and nonusers. We calculated 95% CIs for HIV and syphilis incidence rates using quadratic approximation or exact Poisson methods. We estimated the unadjusted incidence rate ratio (IRR) and adjusted for baseline characteristics with significant between-group differences to estimate the adjusted IRR. We used a univariate generalized estimating equation model with a logit link and robust SEs to assess the adherence over time. The generalized estimating equation model was also used to compare risk behaviors, spinal bone mineral density, quantitative proteinuria, serum creatinine level, and estimated glomerular filtration rate between groups. For different PrEP regimens, the estimated cost of medication based on market prices, incremental cost, the incremental cost per outcome, and total cost per outcome were calculated and summarized for a cost-effectiveness assessment. We used Stata/SE, version 15.0 (StataCorp LLC), for statistical analysis, and 2-sided *P* < .05 was considered statistically significant.

## Results

### Baseline Characteristics of Study Participants

A total of 1530 MSM were included in the analysis (median age, 30 [IQR, 25-37] years). The median age for D-PrEP users was 29 (IQR, 25-35) years; for ED-PrEP users, 29 (IQR, 25-36) years. Most D-PrEP and ED-PrEP users received tertiary education (437 of 520 [84.0%] and 394 of 503 [78.3%], respectively) and had CRAI in the past 3 months (337 of 520 [64.8%] and 319 of 503 [63.4%], respectively). Regarding nonusers, 251 of 507 (49.5%) received tertiary education, and 255 of 507 (50.3%) reported CRAI in the past 3 months. As shown above, D-PrEP and ED-PrEP users were younger and better educated than nonusers. In addition, D-PrEP and ED-PrEP users had higher monthly income (≥¥4000, 252 of 520 [48.5%] and 239 of 503 [47.5%], respectively, vs 170 of 507 [33.5%]) and median HIV Incidence Risk Index score (18 [IQR, 12-22] and 18 [IQR, 11-22], respectively, vs 12 [IQR, 7-18]) and greater prevalence of behaviors associated with HIV risk (eg, median episodes of CRAI, 2 [IQR, 0-5] for both user groups vs 0 [IQR, 0-3]) (all *P* < .001). There was no difference in baseline characteristics between D-PrEP and ED-PrEP users, with the exception of educational level (as shown above; *P* = .02), median number of sex partners (4 [IQR, 2-7] vs 3 [IQR, 2-5]; *P* < .001), anal intercourse with 2 sex partners (442 of 520 [85.0%] vs 400 of 520 [79.5%]; *P* = .02), recreational drug use (282 of 520 [54.2%] vs 208 of 503 [41.4%]; *P* < .001), and history of syphilis infection (58 of 520 [11.1%] vs 38 of 503 [7.5%]; *P* = .03) (Table 1).

**Table 1.** Baseline Characteristics of D-PrEP Users, ED-PrEP Users, and Nonusers

Characteristic	Participant group^a^			*P* value^b^		
	D-PrEP users (n = 520)	ED-PrEP users (n = 503)	PrEP nonusers (n = 507)	D-PrEP users vs nonusers	ED-PrEP users vs nonusers	D-PrEP vs ED-PrEP users
Demographics						
Age, median (IQR), y	29 (25-35)	29 (25-36)	33 (27-43)	<.001	<.001	>.99
Educational level						
High school or less	83 (16.0)	109 (21.7)	256 (50.5)	<.001	<.001	.02
College and greater	437 (84.0)	394 (78.3)	251 (49.5)			
Monthly income, ¥ (US $)						
<4000 (619)	268 (51.5)	264 (52.5)	337 (66.5)	<.001	<.001	.76
≥4000 (619)	252 (48.5)	239 (47.5)	170 (33.5)			
Occupation						
Company employee or civil servant	233 (44.8)	222 (44.1)	165 (32.5)	.05	<.001	.77
Factory worker or farmer	30 (5.8)	31 (6.2)	74 (14.6)			
Freelancer	114 (21.9)	109 (21.7)	133 (26.2)			
Student	77 (14.8)	71 (14.1)	38 (7.5)			
Business services	66 (12.7)	70 (14.0)	97 (19.1)			
Marital status						
Single	279 (53.7)	279 (55.5)	272 (53.6)	.002	.001	.72
Cohabitation with a man	196 (37.7)	176 (35.0)	122 (24.1)			
Married/cohabitation with a woman	30 (5.8)	37 (7.4)	84 (16.6)			
Separated, divorced, or widowed	15 (2.9)	11 (2.2)	29 (5.7)			
Sexual orientation						
Homosexual	412 (79.2)	391 (77.7)	371 (73.2)	.02	.09	.56
Other^c^	108 (20.8)	112 (22.3)	136 (26.8)			
Sexual role with man						
Top	177 (34.0)	170 (33.8)	201 (39.6)	.87	.02	.29
Bottom	158 (30.4)	134 (26.6)	109 (21.5)			
Versatile	180 (34.6)	186 (37.0)	185 (36.5)			
Oral	5 (1.0)	13 (2.6)	12 (2.4)			
Behaviors associated with HIV risk in the past 3 mo						
≥2 Sex partners with anal sex	442 (85.0)	400 (79.5)	310 (61.1)	<.001	<.001	.02
No. of sex partners with anal sex, median (IQR)	4 (2-7)	3 (2-5)	2 (1-4)	<.001	<.001	<.001
Episodes of CRAI, median (IQR)	2 (0-5)	2 (0-5)	0 (0-3)	<.001	<.001	.20
≥1 Sex partner with CRAI	337 (64.8)	319 (63.4)	255 (50.3)	<.001	<.001	.64
Recreational drug use^d^	282 (54.2)	208 (41.4)	128 (25.2)	<.001	<.001	<.001
History of syphilis in the past year	58 (11.1)	38 (7.5)	27 (5)	.02	.70	.03
HIRI-MSM score, median (IQR)	18 (12-22)	18 (11-22)	12 (7-18)	<.001	<.001	>.99
Syphilis by laboratory test at baseline visit	59 (11.3)	43 (8.5)	48 (9.5)	.33	.61	.14

Abbreviations: CRAI, condomless receptive anal intercourse; D-PrEP, daily preexposure prophylaxis; ED-PrEP, event-driven preexposure prophylaxis; HIRI-MSM, HIV Incidence Risk Index for men who have sex with men; PrEP, event-driven preexposure prophylaxis.

^a^ Unless otherwise indicated, data are expressed as number (%) of participants. Percentages have been rounded and may not total 100.

^b^ Calculated using the χ^2^ test for categorical variables, and Wilcoxon rank sum test for continuous variables.

^c^ Includes bisexual and uncertain.

^d^ Includes rush (poppers or alkyl nitrites), MDMA (3,4-methylenedioxymethamphetamine; ecstasy), ice, amphetamines, tramadol hydrochloride, or ketamine hydrochloride.

### HIV Incidence

From December 2018 to November 2020, 904 of 1023 PrEP users (88.4%; overall 1097 person-years) and 447 of 507 nonusers (88.2%; overall 548 person-years) completed the 12-month follow-up. During the study period, 306 PrEP users (29.9%) switched regimens at least once, and 60 (5.9%) switched at least twice. There were 384 episodes of switching (184 from D-PrEP to ED-PrEP and 200 from ED-PrEP to D-PrEP).

There were 35 HIV seroconversions during the 12-month follow-up period, including 2 ED-PrEP users (HIV incidence rate, 0.37 per 100 person-years), 5 D-PrEP users (0.90 per 100 person-years), and 28 nonusers (5.10 per 100 person-years). All 7 PrEP users with seroconversions reported having sexual intercourse without PrEP taken according to prescription between the last HIV-negative visit and the seroconversion visit, and 3 of them had an undetectable serum tenofovir-emtricitabine level at the final visit (eTable 5 in Supplement 2). Overall PrEP use was associated with an 87% reduction in HIV incidence (0.64 vs 5.10 per 100 person-years).

Compared with nonusers, all PrEP users (adjusted IRR, 0.09 [95% CI, 0.04-0.21]), D-PrEP users (adjusted IRR, 0.12 [95% CI, 0.04-0.33]), and ED-PrEP users (adjusted IRR, 0.05 [95% CI, 0.01-0.22]) had significantly lower HIV incidence. There was no difference in HIV incidence between D-PrEP and ED-PrEP users (adjusted IRR, 0.33 [95% CI, 0.06-2.04; *P* = .20]) (Table 2).

**Table 2.** HIV Incidence Among D-PrEP Users, ED-PrEP Users, and PrEP Nonusers

PrEP use	No. with HIV seroconversion	Person-years of follow-up	Incidence rate per 100 person-years (95% CI)	Adjusted IRR (95% CI)^a^	*P* value
Nonusers	28	548.6	5.10 (3.39-7.38)	1 [Reference]	NA
Users					
Overall PrEP	7	1097.3	0.64 (0.26-1.31)	0.09 (0.04-0.21)	<.001
D-PrEP	5	556.0	0.90 (0.29-2.10)	0.12 (0.04-0.33)	<.001
ED-PrEP	2	541.2	0.37 (0.04-1.33)	0.05 (0.01-0.22)	<.001
ED-PrEP vs D-PrEP	2	541.2	0.37 (0.04-1.33)	0.33 (0.06-2.04)	.20

Abbreviations: D-PrEP, daily preexposure prophylaxis; ED-PrEP, event-driven preexposure prophylaxis; IRR, incidence rate ratio; NA, not applicable; PrEP, event-driven preexposure prophylaxis.

^a^ Adjusted for baseline characteristics with significant between-group difference (ie, age, educational level, income, marital status, sex role, number of sexual partners, number of CRAI partners, recreational drug use, and HIV Incidence Risk Index scores for men who have sex with men).

### Adherence to PrEP Regimens and Economic Evaluation

**Figure 2.**
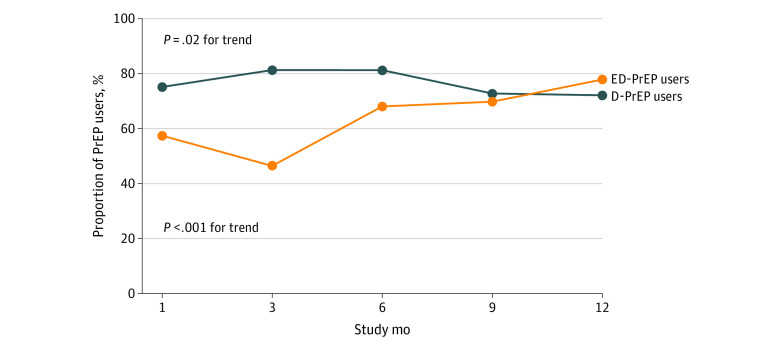
Proportion of Preexposure Prophylaxis (PrEP) Users With 90% or More Self-reported Days of Sexual Intercourse in Which PrEP Was Taken According to Prescription During Follow-up The difference in the rate for total pills taken between daily PrEP (D-PrEP) users and event-driven PrEP (ED-PrEP) users at each follow-up visit.

Figure 2 shows the adherence (defined as the proportion of self-reported days in which intercourse occurred with PrEP use according to prescription of ≥90%) at different points among D-PrEP and ED-PrEP users. Adherence of D-PrEP users decreased over time (from 75.1% to 72.1%; *P* = .02 for trend), whereas the adherence of ED-PrEP users increased over time (from 57.4% to 77.8%; *P* < .001 for trend).

During the study period, the mean (SD) number of tablets consumed by D-PrEP and ED-PrEP users was 6.3 (1.6) and 3.9 (2.4) per week, respectively. The total number of tablets consumed by ED-PrEP users was equivalent to 60.5% (98 574 of 163 299) of those used by D-PrEP users. Given the cost of the tablets ($77-$309 for 30 tablets), the PrEP medication cost ranged from $253 007 to $1 105 312 for ED-PrEP users and $419 134 to $1 681 980 for D-PrEP users during the study period. To prevent 1 new HIV infection, cost for ED-PrEP users was 32% less than that for D-PrEP users (range, $506-$2031 vs $741-$3272) (eTable 6 in Supplement 2).

### Sexual Behaviors and STI Incidence

**Figure 3.**
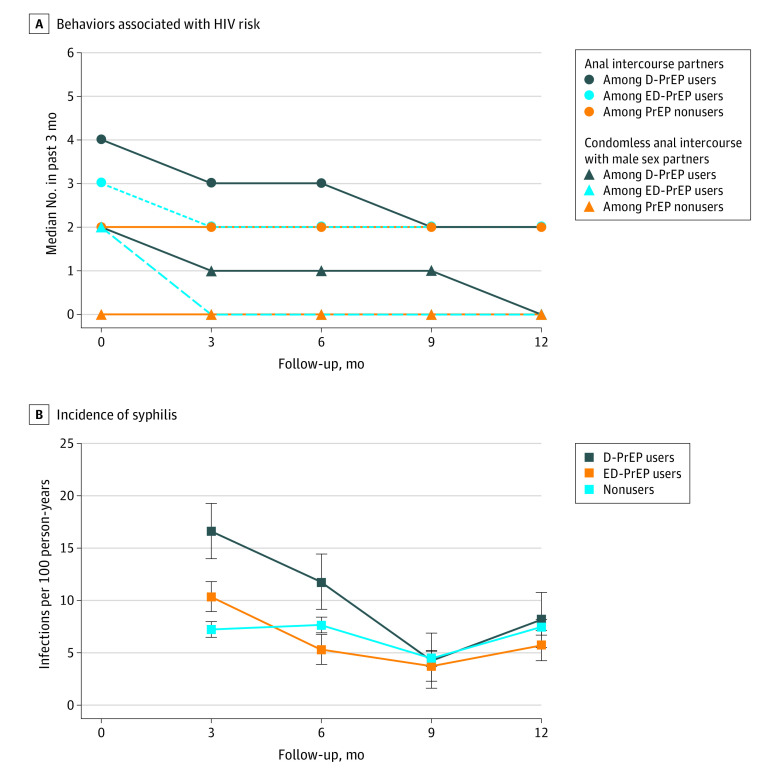
Behaviors Associated With HIV Risk and Incidence of Syphilis During Follow-up of Preexposure Prophylaxis (PrEP) Users and Nonusers Error bars represent 95% CIs. D-PrEP indicates daily PrEP; ED-PrEP, event-driven PrEP.

The median number of sexual partners decreased over time among PrEP users (D-PrEP group, 0.57 [95% CI, 0.45-0.73; *P* < .001 for trend]; ED-PrEP group, 0.68 [95% CI, 0.55-0.83; *P* < .001 for trend]). However, the trend was not observed among nonusers (0.68 [95% CI, 0.33-1.37; *P* = .28 for trend]). The median number of condomless anal intercourse acts decreased over time among D-PrEP users (0.72 [95% CI, 0.57-0.90; *P* = .04 for trend]), but not among ED-PrEP users (1.01 [95% CI, 0.84-1.21; *P* = .89 for trend]) or nonusers (1.01 [95% CI, 0.89-1.13; *P* = .90 for trend]) (Figure 3A). The number of condomless anal intercourse acts was significantly higher among PrEP users than nonusers during follow-up (D-PrEP group: relative risk, 14.37 [95% CI, 1.52-21.55; *P* < .001]; ED-PrEP group: relative risk, 7.46 [95% CI, 3.31-16.79; *P* < .001]) (eTable 7 in Supplement 2).

During the study period, a total of 128 incident syphilis infections were diagnosed, including 56 among the D-PrEP users (10.07 per 100 person-years), 34 among ED-PrEP users (6.28 per 100 person-years), and 38 among nonusers (6.93 per 100 person-years). The overall syphilis incidence was slightly higher among D-PrEP users than nonusers (IRR, 1.45 [95% CI, 0.96-2.20]; *P* = .07). However, such difference was not significant between ED-PrEP users and nonusers (IRR, 0.91 [95% CI, 0.57-1.45]; *P* = .70) (Figure 3B).

### Safety and Adverse Events

No serious adverse events were reported and no PrEP was discontinued because of serious adverse events. Daily PrEP users reported significantly lower prevalence of any adverse events (193 of 520 [37.1%] vs 241 of 503 [47.9%]; *P* < .001) and common adverse events such as dizziness (77 of 520 [14.8%] vs 125 of 503 [24.9%]; *P* < .001), gastrointestinal tract discomfort (129 of 520 [24.8%] vs 192 of 503 [38.2%]; *P* < .001), and fatigue (54 of 520 [10.4%] vs 78 of 503 [15.5%]; *P* = .02) compared with ED-PrEP users. However, the proportion of switching regimens caused by adverse events was lower among ED-PrEP users (1 of 503 [0.2%] vs 11 of 520 [2.1%]; *P* = .004) (eTable 8 in Supplement 2). There was no difference in spinal bone mineral density, quantitative proteinuria, serum creatinine level, estimated glomerular filtration rate, and presence of grade 3 or 4 laboratory abnormalities between D-PrEP and ED-PrEP users (eFigure in Supplement 2).

## Discussion

To our knowledge, this is the first study evaluating D-PrEP and ED-PrEP in real-world settings compared with a parallel group of nonusers. We found that use of D-PrEP and ED-PrEP was associated with lower HIV incidence with a good safety profile. Moreover, ED-PrEP users reported an increasing trend of adherence and consumed fewer pills than D-PrEP users. These data are important to support the feasibility of PrEP in LMICs.

As reported in previous studies,^19^ MSM who were younger, were better educated, and had greater prevalence of behaviors associated with HIV risk were more likely to initiate PrEP; MSM with more sex partners and recreational drug use were more likely to choose D-PrEP than ED-PrEP. Such a pattern is consistent with international PrEP guidelines^11^ recommending that MSM with frequent sexual activity should choose D-PrEP.

Despite the much higher prevalence of behaviors associated with HIV risk among PrEP users compared with the nonusers, PrEP use was associated with a much lower risk of HIV infection. The observed outcome of D-PrEP use in our study was similar to that reported in randomized clinical trials^2,4^ and was slightly higher than that in a demonstration project in Africa.^20^ Consistent with previous open-label prospective cohorts,^8,9,10^ the observed outcomes of D-PrEP and ED-PrEP use were similar. Incident HIV seroconversion among PrEP users could be explained by suboptimal adherence. Given that ED-PrEP had lower cost to prevent new HIV infection, China and other LMICs may consider ED-PrEP as an alternative to D-PrEP, which is potentially more cost-effective than only implementing D-PrEP.^1,21^

Consistent with previous studies,^10,22^ D-PrEP users had better adherence than ED-PrEP users. Interestingly, the trends in adherence were different between D-PrEP and ED-PrEP users. In line with a study from Brazil,^23^ adherence decreased over time among D-PrEP users. In contrast, although adherence was relatively low among ED-PrEP users at month 1 (57.4%), it increased over time and reached 77.8% at month 12. Users might take some time to adjust the dosing schedule of ED-PrEP according to their sexual behavior patterns. After ED-PrEP users become familiar with the dosing schedule, they might be less likely to miss doses, because PrEP use was closely associated with sexual behaviors. Given the increasing adherence, the cost for long-term adherence support among ED-PrEP users may also be lower than among D-PrEP users.

The prevalence of grade 3 or 4 adverse events was slightly lower than in previous studies^24,25^ and did not differ between groups. These findings suggest that both D-PrEP and ED-PrEP are safe for Chinese MSM. However, the prevalence of dizziness and gastrointestinal tract discomfort was higher among ED-PrEP users than D-PrEP users. Previous studies^26^ have suggested the presence of a priming syndrome after PrEP initiation, which typically peaks 1 month after PrEP initiation and declines to baseline levels 2 months later. Given the interruption of medication use and the doubling of the dose before sex, the duration of start-up syndromes may be longer among ED-PrEP users compared with D-PrEP users, which would lead to more self-reported adverse events.

In contrast to the findings of previous demonstration projects,^27^ we did not observe an increase in HIV-related risk behaviors among D-PrEP or ED-PrEP users. Because PrEP users need to receive regular STI screening, more asymptomatic patients with STIs would be identified and linked to early treatment. Future studies should look at the potential impact of PrEP scale-up on STI incidence at the population level among Chinese MSM.

### Limitations

This study also has some limitations. First, sexual risk behaviors were self-reported. Participants might underreport such behaviors owing to social stigma. Such reporting bias might apply to both D-PrEP and ED-PrEP users. Second, adherence might be overestimated owing to the free availability of PrEP and incentives for participating in this study. Third, consistent with previous real-world research,^19,28^ most PrEP users were well educated. Future research should focus on implementation and outcomes of PrEP use among less-educated MSM. Furthermore, the decrease in risk behaviors and incident syphilis infection after month 9 follow-up might be associated with the COVID-19 outbreak and its strict control measures in China.

## Conclusions

The findings of this study suggest that D-PrEP and ED-PrEP regimens were both associated with lower incidence of HIV and a good safety profile among high-risk MSM in China. Event-driven PrEP may be considered an alternative option to D-PrEP for MSM in LMICs such as China.

## Notes

Supplement 1.
Trial Protocol

Supplement 2. **eTable 1.** Procedures of Clinic Visits of the CROPrEP Project **eTable 2.** Laboratory Procedures **eTable 3.** Threshold Values for Grade 3 or 4 Laboratory Abnormality **eTable 4.** HIRI-MSM Risk Index **eTable 5.** MSM Who Initiated PrEP and Nonusers With Incident Seroconversion During the Study Period **eTable 6.** Economic Evaluation of Tenofovir-Emtricitabine for PrEP in Preventing HIV Infection Among Daily and Event-Driven Dosing Regimen **eTable 7.** Overall Comparison of Sexual Behavior per 3 Months During 12-Month Follow-up Between Daily PrEP Users, Event-Driven PrEP Users, and PrEP Nonusers **eTable 8.** Occurrence of Adverse Events Among Daily and Event-Driven PrEP Users **eFigure.** Changes in Spinal BMD, Quantitative Proteinuria, Serum Creatinine, and Creatinine Clearance Among D-PrEP and ED-PrEP Users During Follow-up

Supplement 3.
Nonauthor Collaborators

Supplement 4.
Data Sharing Statement
