# Supplementary material for: Association of HIV Preexposure Prophylaxis Use With HIV Incidence Among Men Who Have Sex With Men in China: A Nonrandomized Controlled Trial
Source: JAMA Netw Open. 2022 Feb 16;5(2):e2148782. doi: 10.1001/jamanetworkopen.2021.48782 (PMC8851305; doi:10.1001/jamanetworkopen.2021.48782)
Supplement: Supplement 1. — Trial Protocol [file jamanetwopen-e2148782-s001.pdf]

Protocol for a multicenter, real-world study of HIV pre-exposure prophylaxis among men who have sex with men in China (*CROPrEP*)

Research objectives

The objectives of the *CROPrEP* project are to assess among HIV-negative Chinese MSM:

1. The real-life effectiveness of daily or event-driven PrEP use
2. Adherence to PrEP
3. Safety and tolerability
4. The potential effects of PrEP use on sexual behaviors and incidence of sexually transmitted infections (STIs)

Methods/Design

Overview of study design

As shown in Fig 1, the *CROPrEP* project is a multicenter, real-world, prospective cohort study with two arms of daily or event-driven TDF/FTC as PrEP among HIV-negative MSM at high risk of HIV infection in China. This study is being conducted in four metropolitan Chinese cities, which have moderate to high prevalence of HIV among MSM: Shenyang, Beijing, Shenzhen, and Chongqing. In total, 1023 eligible participants will be permitted to choose between daily or event-driven PrEP, based on their preference. Participants will be prospectively followed for 12 months, with quarterly clinic visits, and online weekly notes. The PrEP intervention, in combination with a package of prevention services, will be provided to participants (e.g., free adherence supporting reminders and counseling, sexual health education, panel management by peer counselors, and referrals to relevant clinics for individuals who test positive for either HIV or syphilis). These four study centers have also established

an expanding cohort study of 500 HIV-1-negative MSM for epidemic surveillance and estimation, which can be considered a natural background control for PrEP users, to evaluate the efficacy of PrEP use.

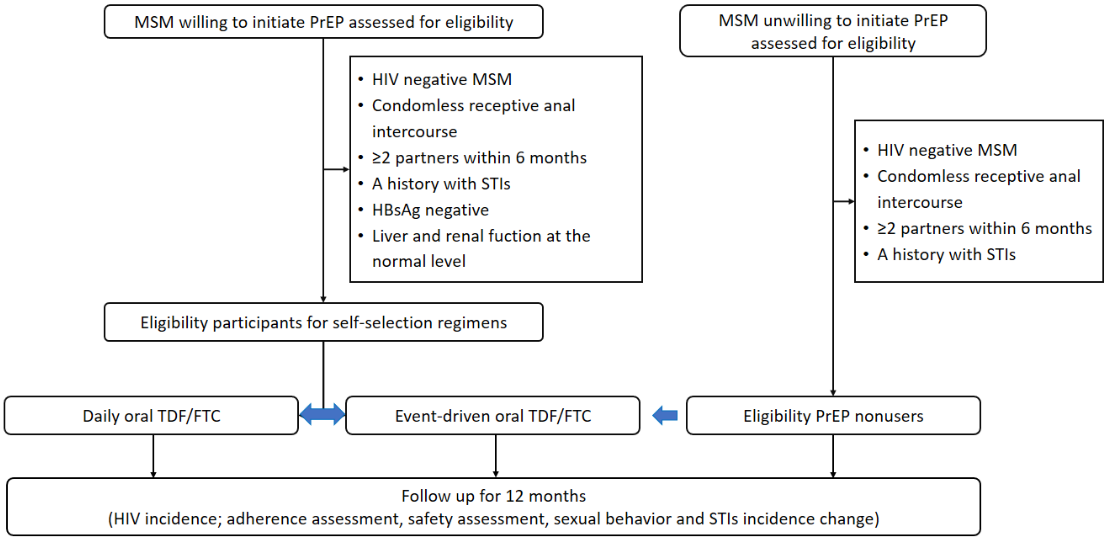

Fig. 1 Flow chart of study design

PrEP users will be permitted to switch their PrEP regimen. PrEP nonusers can initiate PrEP at any time if they want.

Sample size

Given the well-documented protective effect of PrEP, the *CROPrEP* project is considered a demonstration project, and the sample size is not determined by power calculations. Based on preliminary survey data of acceptance among MSM in China, the sample size was increased as far as possible, on the basis of fully considering the population of each research center, and a largest sample size of 1000 chosen.

Project promotion and recruitment of participants

Participant recruitment is performed by both online and offline methods. Online methods rolled out through accounts managed by each research center include advertising on public social platforms, such as Weibo and WeChat. Offline recruitment methods include the following: (1) clinic-based sampling:

each study center collaborates with the local CDC and clinic staff will recruit MSM who already attend voluntary counseling and testing clinics, are at high risk of HIV infection, and are willing to participate in the *CROPrEP* after learning about the study; (2) community-based sampling: each study center collaborates with local MSM community-based organizations (CBOs) to recruit MSM in the community; (3) venue-based sampling: eligible MSM will be recruited from suitable venues (e.g., MSM parks, bars, clubs, and bathhouses) through outreach activities; and (4) peer referrals: potential participants will be encouraged to recruit their friends who are MSM or their male partners to participate in this study. All candidates will be preregistered and then invited for a screening visit at the local study center on the principle of competitive enrollment. Prior to the eligibility assessment for the study, written informed consent will be obtained from all participants. PrEP nonusers will be recruited using the same way as PrEP users.

Participant eligibility and enrollment

As shown in Table 1, the inclusion and exclusion criteria will be used to assess the eligibility of participants in the *CROPrEP* project. These criteria are established by an expert group of researchers, clinicians, and CBO representatives, based on international guidelines on PrEP and the behavior characteristics of MSM in China.

Eligibility will be assessed for ongoing comprehensive screening at a clinic, including laboratory testing for antibodies and RNA for HIV, syphilis, hepatitis B virus surface antigen (HBsAg), bone mineral density (BMD), renal and liver function, clinical evaluation with family or personal medical history, concomitant treatment by an experienced physician, and a self-administered questionnaire, to assess sociodemographic factors, current sexual behavior, substance use, and social and psychological information, on their mobile phones. Confirmed eligible participants will be invited to return to the

clinic within a week to choose a regimen and receive a supply of TDF/FTC and detailed instructions for PrEP use.

Table 1 Inclusion and exclusion criteria for participants in the *CROPrEP* project

Inclusion criteria

- Aged 18–65 years old*
- Born to male sex*
- Test results demonstrate HIV negative*
- Had sex with a man in the last 6 months*
- **Behavioral eligibility criteria***

  Participants of male sex at birth and who have sex with men, reporting at least one criterion associated with high risk for HIV infection in the 6 months prior to enrolment as follows:

  - Unprotected (condom-less) receptive anal intercourse with male partners
  - More than two male partners (regardless of condom use and HIV serostatus)
  - Reported STI, such as syphilis, HSV-2, gonorrhea, chlamydia, chancroid, or lymphogranuloma venereum
  - Reported a history of post-exposure prophylaxis

  Note: Individuals in a monogamous relationship with an HIV-1 seronegative partner or a virologically suppressed HIV-1+ partner for > 1 year will not be eligible for participation.
- Through comprehensive physical examination, including routine urine examinations, hepatic and renal function tests, blood glucose and lipids, and BMD; no serious liver or kidney dysfunction and negative for HBs antigen, without serology indicating osteoporosis, and other indicators are normal
- Able and being willing to sign written informed consent and participate in the study as procedures require*
- Chinese citizens*

Exclusion criteria

- HIV-1 infected, or having clinical signs or symptoms consistent with acute viral infection*
- Atopic individual or allergic to the ingredients of the experimental drug or ART*
- Having serious chronic disease, including metabolic diseases (such as diabetes), neurological, and psychiatric disorders
- Weight < 40 kg or > 140 kg*
- Having osteoporosis: aged ≥ 50 years with BMD T-score ≤ –2.5; aged < 50 years with BMD Z-score ≤ –2 and fragility fracture
- Mental health issues which may compromise participant adherence or safety, including memory loss, cognitive impairment, intellectual disability, or communication disorders
- Currently, or 30 days prior to enrolment, taking interferon, interleukin, or other immunoregulators*

- Currently taking products containing antiretrovirals*
- Participating in another research study related to HIV and antiretroviral therapy or other intervention study*

PrEP participants need to meet all eligibility criteria;
*Eligibility criteria for PrEP nonusers.

Drug regimen and provision

The PrEP used in the *CROPrEP* project is Truvada®; one film-coated pill contains 200 mg of FTC and 300 mg of TDF. Participants will receive free Truvada and can choose between taking PrEP on a daily basis, or according to an event-driven regimen, before and after anal sex. The daily dosing regimen is one Truvada pill every 24 h. The event-driven regimen is two Truvada pills 2–24 h before sexual intercourse (or one pill if the last medication was taken 1 to 6 days ago), a pill every 24 h from the first drug intake during the period of sexual activity, including after the last sexual intercourse, and one final Truvada pill approximately 24 h later. PrEP users will be permitted to switch their PrEP regimen during the study period. All PrEP users followed such advice. PrEP nonusers will be followed up at the same clinic. They can initiate PrEP at any time if they want.

Study visits

Each PrEP participant will be followed up for 12 months and will experience a total of six clinic visits at centers (including screening (baseline) and follow-up (FU) at 1, 3, 6, 9, and 12 months) (Table 2). PrEP nonusers should complete four quarterly follow-up visits for a year. The visit window for this study is 7 days. For each clinic visit, adverse events and concomitant medication will be documented and HIV/STI testing and monitoring of biological indices for PrEP-related toxicity performed by experienced physicians. Participants will be instructed to complete an online self-administered questionnaire to assess behaviors, psychological status, adherence, side effects, and attitude towards PrEP use in a separate private interview room. Dry blood spots and blood samples will be collected

from participants who report that they have taken PrEP during the follow-up interval for TDF/FTC concentration and HIV/STI testing, alongside other clinical evaluations. Participants will be required to return the leftover TDF/FTC to the clinic in exchange for a refill to cover their needs until the next clinic visit.

Online weekly notes will be sent to the mobile phones of participants weekly, to feedback their short-term pill use and sexual practices. Participants will be instructed to complete the message every week, to limit recall bias.

During the follow-up period, researchers will benefit from leveraging CBOs and the internet to strengthen group- and individual-level supervision of adherence to medication and cohort management. This multifaceted strategy will include a panel of CBO counselors, providing interactive peer counseling focusing on study retention; a Short Message Service application, providing routine online medication reminders and follow-up visit reminders, along with a live chat; and clinicians and study staff, providing one-on-one personalized compliance support, counseling, and cohort maintenance.

Table 2 Schedule of the *CROPrEP* project

**Procedures**	**Screening/ Enrollment**	**FU 1 m±7d[#]**	**FU 3 m±7d**	**FU 6 m±7d**	**FU 9 m±7d**	**FU 12 m±7d**
Informed consent[*]	×					
Self-administrated questionnaire	×	×	×	×	×	×
Interviews						
Relevant medical history	×					
Current/concomitant medication	×	×	×	×	×	×
Adverse events or side effects		×	×	×	×	×
Medication return and allocation	×	×	×	×	×	×
HIV/STIs testing[*]						
HIV screening	×	×	×	×	×	×
HIV Western Blot[a]	×	×	×	×	×	×
HIV RNA Pooling PCR[b]	×	×	×	×	×	×
HIV resistance testing[c]						
HIV-1 viral load[c]						
Syphilis	×	×	×	×	×	×

Safety assessment						
Routine blood tests	×	×	×	×	×	×
Routine urine tests	×	×	×	×	×	×
Liver functions tests	×	×	×	×	×	×
Renal functions tests	×	×	×	×	×	×
Blood glucose and lipids	×	×	×	×	×	×
Bone mineral density	×			×		×
Hepatitis B virus	×			×		×
Adherence lab assessment						
Blood drug level testing		×	×	×	×	×

Abbreviations: FU, follow-up; m, month(s); d, day. [*] Items that PrEP nonusers need to complete. [#] PrEP nonusers do not need to attend this follow-up visit. [a]Anyone who screens positive for HIV will have the results confirmed by western blotting. [b]Anyone who screens negative for HIV will have the results confirmed by HIV RNA pooling PCR test. [c]Anyone who tests positive for HIV will have their samples further tested for HIV resistance and viral load.

Laboratory Procedures

As summarized in Table 3, during each clinical visit, participants will be asked to provide blood and urine specimens for monitoring of HIV infection, STIs, and objective measures for adherence and safety of PrEP use. Analysis of blood TDF/FTC concentrations will be performed in the Key Laboratory of AIDS Immunology of National Health Commission, Department of Laboratory Medicine, The First Affiliated Hospital, China Medical University, in Shenyang, China. All other testing will be conducted by nationally certified laboratories, located at each research center. Dry blood spots and serum samples will be collected at every visit for TDF/FTC drug level testing.

If a participant is found to have seroconverted to HIV, his participation in the study will be terminated immediately and he will be called for a final visit. At the final visit, study staff will collect unused pills, conduct resistance and viral load testing, and provide referrals to an HIV clinic for treatment. The study staff will report each case of seroconversion to the sponsor.

Table 3 Laboratory Parameters

Laboratory Parameter	Test

HIV	HIV serostatus is evaluated by ELISA (InTec Products Company, Xiamen, China), and confirmed with an HIV-1/2 western blot (HIV Blot 2.2 WBTM, Genelabs Diagnostics, Singapore). The results of anyone who screens negative for HIV will be confirmed by HIV RNA pooling PCR test (COBAS AmpliPrep /COBAS TaqMan, Roche)
HIV resistance	RNA sequencing
HIV viral load testing	COBAS AmpliPrep /COBAS TaqManv (Roche)
Syphilis	RPR (Shanghai Rongsheng, Shanghai, China) and TPPA (Fujirebio Inc, Tokyo, Japan)
Hepatitis B	HBsAg (Vitros 5600)
HSV-2	IgM-HSV-2, IgG-HSV-2 (Beier Bioengineering, Beijing, China)
Biochemistry	AST, ALT, total bilirubin, creatinine, creatinine clearance, serum phosphate, blood glucose, and lipids (Roche)
Routine blood tests	Full blood count: hemoglobin, leucocytes, platelets; differential count: absolute neutrophil count, absolute lymphocyte count (Mindray BC-5800)
Routine urine tests	Proteinuria (Mindray EH-2080)
Blood drug level testing	LC-MS (AB SCIEX API 6500+)

Abbreviations: ELISA, enzyme-linked immunosorbent assay; RPR, rapid plasma reagin; TPPA, *Treponema pallidum* particle agglutination assay; HSV-2, Herpes simplex virus 2; HBsAg, Hepatitis B surface antigen; AST, aspartate transferase; ALT, alanine transferase; LC-MS, Liquid chromatography-mass spectrometry.

Safety assurance

All PrEP users should receive safety assurance at each follow-up. Safety of TDF/FTC will be monitored by laboratory ratings, based on vital signs and adverse events recorded on a paper case report form at each clinic visit, and the results of causal association and severity assessment will be independently evaluated by two physicians. Any reported adverse events will be clinically tracked until they are restored or stabilized. Serious adverse events are life-threatening events, including HIV

seroconversions that must be reported to the sponsor within one business day, and the lead ethics committee within 72 h.

Data collection and quality assurance

The results from both types of data collection will be triangulated to assess various end points. A specialized platform for questionnaire collection will be used to collect self-administered, structured questionnaires to assess socio-demographic information, perceived skills, willingness, impact of PrEP use on behaviors and mental status, self-reported adherence, and side effects. All examination results will be interpreted and organized into structured data by professional physicians. For unstructured data, such as medical records and drug release records, core information on adverse events, medications, and tablet counts will be extracted by trained staff. Over the course of the study, we will establish and strictly operate a system of quality control to ensure the integrity, validity, and authenticity of data (Fig 2). Besides, data inspection companies are employed as independent from investigators and the sponsor for auditing trial conduct.

Study end points

As shown in Table 4, primary and secondary end points will be pursued to determine the feasibility and operationalization of PrEP implementation in China.

Adherence to PrEP will be measured from different perspectives: drug concentration testing, self-reported adherence via questionnaires and weekly messages, self-report to clinician, dispensing and return records, and drug level assessment.

Table 4 End points of the *CROPrEP* project

1. The real-life effectiveness of daily or event-driven PrEP use:

— HIV incidence among individuals using different PrEP regimens, compared with that of non-PrEP users randomly selected by propensity score from local expanding cohort studies of

HIV-negative MSM

— The rate of viral genotype resistance among individuals who seroconvert to HIV during the study

period

2. Adherence to PrEP:

— Number, proportion and patterns of prescribed doses taken and missed according to self—reported adherence via online questionnaires and weekly messages, reported pill use, and pill counts

— Testing of FTC/TDF drug concentration in blood samples

— Percentage of participants who switch regimens and their reasons

3. Safety and tolerability:

— Rate of side effects or adverse events related to PrEP use

— Rate of adverse events related to discontinuation of PrEP or switching PrEP regimens

3. The potential effects of PrEP use on sexual behaviors and incidence of sexually transmitted infections (STIs):

— Potential changes in the number of sexual partners and the numbers of casual or steady partners

— Potential changes in condom use

— Potential changes in the incidence of syphilis

— Changes in the use of sexual networks

— Changes in perception of sexual well-being during sexual intercourse

Data analysis

The sociodemographic characteristics of the study population will be analyzed using descriptive statistics (mean/standard deviation, or median/interquartile range) and distributions compared using χ^2 or Fisher’s exact tests, as appropriate. Sexual behaviors, medication adherence, and risk of HIV seroconversion will be analyzed using generalized estimating equation models. All computations will be conducted using STATA version 15.0 (IBM Corp).
