# Supplementary material for: Association of HIV Preexposure Prophylaxis Use With HIV Incidence Among Men Who Have Sex With Men in China: A Nonrandomized Controlled Trial
Source: JAMA Netw Open. 2022 Feb 16;5(2):e2148782. doi: 10.1001/jamanetworkopen.2021.48782 (PMC8851305; doi:10.1001/jamanetworkopen.2021.48782)
Supplement: Supplement 2. — eTable 1. Procedures of Clinic Visits of the CROPrEP Project eTable 2. Laboratory Procedures eTable 3. Threshold Values for Grade 3 or 4 Laboratory Abnormality eTable 4. HIRI-MSM Risk Index eTable 5. MSM Who Initiated PrEP and Nonusers With Incident Seroconversion During the Study Period eTable 6. Economic Evaluation of Tenofovir-Emtricitabine for PrEP in Preventing HIV Infection Among Daily and Event-Driven Dosing Regimen eTable 7. Overall Comparison of Sexual Behavior per 3 Months During 12-Month Follow-up Between Daily PrEP Users, Event-Driven PrEP Users, and PrEP Nonusers eTable 8. Occurrence of Adverse Events Among Daily and Event-Driven PrEP Users eFigure. Changes in Spinal BMD, Quantitative Proteinuria, Serum Creatinine, and Creatinine Clearance Among D-PrEP and ED-PrEP Users During Follow-up [file jamanetwopen-e2148782-s002.pdf]

Supplementary Online Content

Wang H, Wang Z, Huang X, et al; China Real-World Oral Intake of PrEP (CROPrEP) Study Team. Association of HIV preexposure prophylaxis use with HIV incidence among men who have sex with men in China: a nonrandomized controlled trial. *JAMA Netw Open*. 2022;5(2):e2148782. doi:10.1001/jamanetworkopen.2021.48782

eTable 1. Procedures of Clinic Visits of the CROPrEP Project

eTable 2. Laboratory Procedures

eTable 3. Threshold Values for Grade 3 or 4 Laboratory Abnormality

eTable 4. HIRI-MSM Risk Index

eTable 5. MSM Who Initiated PrEP and Nonusers With Incident Seroconversion During the Study Period

eTable 6. Economic Evaluation of Tenofovir-Emtricitabine for PrEP in Preventing HIV Infection Among Daily and Event-Driven Dosing Regimen

eTable 7. Overall Comparison of Sexual Behavior per 3 Months During 12-Month Follow-up Between Daily PrEP Users, Event-Driven PrEP Users, and PrEP Nonusers

eTable 8. Occurrence of Adverse Events Among Daily and Event-Driven PrEP Users

eFigure. Changes in Spinal BMD, Quantitative Proteinuria, Serum Creatinine, and Creatinine Clearance Among D-PrEP and ED-PrEP Users During Follow-up

This supplementary material has been provided by the authors to give readers additional information about their work.

© 2022 Wang H et al. *JAMA Network Open*.

eTable 1. Procedures of Clinic Visits of the CROPrEP Project

**Procedures**	**Baseline**	**FU 1m±7d**	**FU 3m±7d**	**FU 6m±7d**	**FU 9m±7d**	**FU 12m±7d**
Informed consent	x					
Self-administered online survey [a]	x	x	x	x	x	x
Interviews conducted by experienced physicians						
Relevant medical history	x	x	x	x	x	x
Current/concomitant medication	x	x	x	x	x	x
Adverse events or side-effects		x	x	x	x	x
Truvada ® dispense/refill	x	x	x	x	x	x
Return leftover Truvada ®		x	x	x	x	x
**HIV testing**						
HIV screening	x	x	x	x	x	x
HIV western blot [b]	x	x	x	x	x	x
HIV RNA Pooling PCR [c]	x	x	x	x	x	x
HIV resistance testing [d]	x	x	x	x	x	x
HIV viral load [d]	x	x	x	x	x	x
**Sexually transmitted infections (STI) testing**						
Syphilis	x	x	x	x	x	x
**Safety assessment**						
Routine blood tests	x	x	x	x	x	x
Routine urine tests	x	x	x	x	x	x
Liver functions tests	x	x	x	x	x	x
Renal function tests	x	x	x	x	x	x
Blood glucose and lipids	x	x	x	x	x	x
Bone mineral density	x	x	x	x	x	x
Hepatitis B virus testing	x	x	x	x	x	x
Adherence lab assessment		x	x	x	x	x
Serum TDF/FTC testing		x	x	x	x	x

[a] Participants scanned a Quick Response (QR) code to complete a self-administered online baseline survey on site. The survey collected sociodemographic, sexual behaviors, substance use, and social and psychological information. Participants completed online follow-up surveys using the same manners as that of the baseline to assess their behaviors, psychological status, adherence, side-effects, and attitudes related to PrEP in separate rooms with privacy protected.

[b] Anyone who screens positive for HIV will have the results confirmed by western blot

[c] Anyone who screens negative for HIV will have the results confirmed by HIV RNA pooling PCR test

[d] Anyone who confirms as HIV positive by western blot will have their sample tested for HIV resistance and viral load.

© 2022 Wang H et al. *JAMA Network Open*.

eTable 2. Laboratory Procedures

Laboratory parameter	Tests
HIV	HIV serostatus is evaluated by ELISA (InTec Products Company, Xiamen, China) and confirmed with an HIV-1/2 western blot (HIV Blot 2.2 WBTM, Genelabs Diagnostics, Singapore).<br>The results of anyone who screens negative for HIV will be confirmed by HIV RNA pooling PCR test (COBAS AmpliPrep /COBAS TaqMan, Roche)
HIV resistance	RNA sequencing
HIV viral load/ HIV plooing RNA	Roche Cobas Ampliprep/Taqman
Syphilis	RPR (Shanghai Rongsheng, Shanghai, China) and TPPA (Fujirebio Inc., Tokyo, Japan)
Routine blood tests	Full blood count: hemoglobin, leucocytes, platelets; differential count: absolute neutrophil count, absolute lymphocyte count (Mindray BC-5800)
Routine urine tests	Proteinuria (Mindray EH-2080)
Serum TDF/FTC testing	LC-MS (AB SCIEX API 6500+)

ELISA=Enzyme-linked immunosorbent assay; PRP=Rapid plasma regain; TPPA=*Treponema pallidum* particle assay; LC-MS=liquid chromatography-mass spectrometry.

eTable 3. Threshold Values for Grade 3 or 4 Laboratory Abnormality

Biochemical	Indexes values
Increased alanine aminotransferase	> 200 U/L
Increased aspartate aminotransferase	> 200 U/L
Hyperglycemia, fasting	> 13·89 mmol/L
Increased LDL, fasting	> 4·9 mmol/L
Glycosuria	> 2+

© 2022 Wang H et al. *JAMA Network Open*.

eTable 4. HIRI-MSM Risk Index

Items	Level	Scores*
How old are you today (yrs)?	<18 years	0
	18–28 years	8
	29–40 years	5
	41–48 years	2
	≥49 years	0
How many men have you had sex with in the last 6 months?	>10 male partners	7
	6–10 male partners	4
	0–5 male partners	0
In the last 6 months, how many times did you have receptive anal sex (you were the bottom) with a man?	1 or more times	10
	0 times	0
How many of your male sex partners were HIV positive?	>1 positive partner	8
	1 positive partner	4
	<1 positive partner	0
In the last 6 months, how many times did you have insertive anal sex (you were the top) with a man who was HIV positive?	5 or more times	6
	0 times	0
In the last 6 months, have you used methamphetamines such as crystal or speed?	Yes	5
	No	0
In the last 6 months, have you used poppers (amyl nitrate)?	Yes	3
	No	0

* If total score is 10 or greater, evaluate for PrEP or other intensive HIV prevention services.

© 2022 Wang H et al. *JAMA Network Open*.

eTable 5. MSM Who Initiated PrEP and Nonusers With Incident Seroconversion During the Study Period

No.	PrEP regimen	Final visit	Final visit date	Serum TDF/FTC level	Self-reported medication adherence
1	D-PrEP	1m	2019-8-5	TDF: 107ng/ml<br>FTC: 116ng/ml	Reported condomless receptive anal intercourse with casual sex partner five days after stop taking PrEP.
2	D-PrEP	9m	2020-1-13	TDF: Not detected<br>FTC: Not detected	Self-reported missing doses in 30 days and 10 sexual acts uncovered by PrEP in the past three months
3	D-PrEP	1m	2019-6-19	TDF: 20·4ng/ml<br>FTC: 42·6ng/ml	Follow-up was delayed for one month, intermittent medication, self-reported missing doses in 30 days and have 15 condomless receptive in the past two months
4	D-PrEP	3m	2019-10-28	TDF: 181ng/ml<br>FTC: 453ng/ml	Self-reported missing doses in 10 days and two sexual acts uncovered by PrEP in the past three months
5	D-PrEP	9m	2020-8-7	TDF: Not detected<br>FTC: Not detected	Self-reported missing doses in 90 days and more than 20 sexual acts uncovered by PrEP in the past three months
6	ED-PrEP	12m	2020-5-22	TDF: Not detected<br>FTC: Not detected	Self-reported missing doses in 25 days and three sexual acts uncovered by PrEP in the past three months
7	ED-PrEP	1m	2019-6-22	TDF: 34·8ng/ml<br>FTC: 22·9ng/ml	Reported 9 episodes of sexual behaviors after enrollment, missed 6 doses of PrEP before sexual behaviors, no missing doses 24h and 48h after sexual behaviors
8	PrEP nonusers	m3	2019/3/27	N/A	N/A
9	PrEP nonusers	m3	2019/6/1	N/A	N/A
10	PrEP nonusers	m3	2019/9/18	N/A	N/A
11	PrEP nonusers	m3	2019/10/23	N/A	N/A
12	PrEP nonusers	m3	2019/11/19	N/A	N/A
13	PrEP nonusers	m6	2019/8/7	N/A	N/A
14	PrEP nonusers	m6	2020/6/5	N/A	N/A
15	PrEP nonusers	m9	2020/9/4	N/A	N/A
16	PrEP nonusers	m12	2019/9/26	N/A	N/A
17	PrEP nonusers	m12	2020/1/11	N/A	N/A
18	PrEP nonusers	m12	2020/3/12	N/A	N/A
19	PrEP nonusers	m12	2020/4/26	N/A	N/A
20	PrEP nonusers	m12	2020/7/31	N/A	N/A

© 2022 Wang H et al. *JAMA Network Open*.

21	PrEP nonusers	m12	2020/8/25	N/A	N/A
22	PrEP nonusers	m12	2020/9/11	N/A	N/A
23	PrEP nonusers	m12	2020/9/11	N/A	N/A
24	PrEP nonusers	m12	2020/9/13	N/A	N/A
25	PrEP nonusers	m12	2020/9/24	N/A	N/A
26	PrEP nonusers	m12	2020/10/12	N/A	N/A
27	PrEP nonusers	m12	2020/10/16	N/A	N/A
28	PrEP nonusers	m12	2020/10/26	N/A	N/A
29	PrEP nonusers	m12	2020/11/5	N/A	N/A
30	PrEP nonusers	m12	2020/11/11	N/A	N/A
31	PrEP nonusers	m12	2020/11/11	N/A	N/A
32	PrEP nonusers	m12	2020/11/12	N/A	N/A
33	PrEP nonusers	m12	2020/11/13	N/A	N/A
34	PrEP nonusers	m12	2020/11/13	N/A	N/A
35	PrEP nonusers	m12	2020/11/20	N/A	N/A

eTable 6. Economic Evaluation of Tenofovir-Emtricitabine for PrEP in Preventing HIV Infection Among Daily and Event-Driven Dosing Regimen

**Regimen**	**Cost (US $)***	**HIV seroconversions**	**Outcomes † (HIV negative)**	**Incremental cost**	**incremental outcome**	**Incremental cost per outcome**	**Cost per person (US $)**
ED-PrEP	253,007-1015,312	2	501	Ref.			506-2,031
D-PrEP	419,134-1681,980	5	515	166,127-666,668	14	11,889-47,712	741-3,272

*Total drug expenditures were calculated at $77-309 per 30 tablets for the total tablet consumption in D-PrEP users and ED-PrEP users (98,574 and 163,299 tablets, respectively). † Outcomes is equal to the number of participants enrolled minus the number of HIV seroconversions among different regimens.

© 2022 Wang H et al. *JAMA Network Open*.

eTable 7 Overall Comparison of Sexual Behavior per 3 Months During 12-Month Follow-up Between Daily PrEP Users, Event-Driven PrEP Users, and PrEP Nonusers

	D-PrEP users			ED-PrEP users	
	RR (95%CI)	p value		RR (95%CI)	p value
Sexual partners, n	5.73 (1.52-21.55)	0.01		0·94 (0.33-2.63)	0.90
Condomless sex acts, n	14.37 (7.40-27.88)	<0.001		7·46 (3.31-16.79)	<0.001

The PrEP nonusers were used as the reference group. aRR=unadjusted relative ratio. PrEP=pre-exposure prophylaxis.

eTable 8. Occurrence of Adverse Events Among Daily and Event-Driven PrEP Users

Adverse events	D-PrEP users (n=520)	ED-PrEP users (n=503)	*P* values
Any adverse events	193 (37.1%)	241 (47.9%)	<.001
Change regiments of drug because of adverse event	11 (2.1%)	1 (0.2%)	.004
Discontinuation of drug because of adverse event	3 (0.6%)	1 (0.2%)	.64
Serious adverse event*	0 (0.0%)	0 (0.0%)	NA
Common adverse events (≥10% in either group)			
Dizziness	77 (14.8%)	125 (24.9%)	<.001
Headache	33 (6.3%)	48 (9.5%)	.06
Gastrointestinal discomfort (diarrhea, stomachache)	129 (24.8%)	192 (38.2%)	<.001
Fatigue	54 (10.4%)	78 (15.5%)	.02
Decreased appetite	35 (6.7%)	42 (8.3%)	.33
Grade 3 or 4 laboratory abnormality (≥1% in either group)			
Increased alanine aminotransferase§	7 (1.3%)	6 (1.2%)	.83
Increased aspartate aminotransferase§	5 (1.0%)	3 (0.6%)	.76
Hyperglycemia, fasting§	3 (0.6%)	1 (0.2%)	.64
Increased LDL, fasting§	11 (2.1%)	12 (2.4%)	.77
Glycosuria§	27 (5.2%)	27 (5.4%)	.90

*The serious adverse events were appendicitis, suicidal ideation, acute kidney injury, cellulitis, pneumonia, suicide attempt.
§Threshold values for the defined concentrations are in the eTable 8 in the Supplement.

© 2022 Wang H et al. *JAMA Network Open*.

eFigure. Changes in Spinal BMD, Quantitative Proteinuria, Serum Creatinine, and Creatinine Clearance Among D-PrEP and ED-PrEP Users During Follow-up

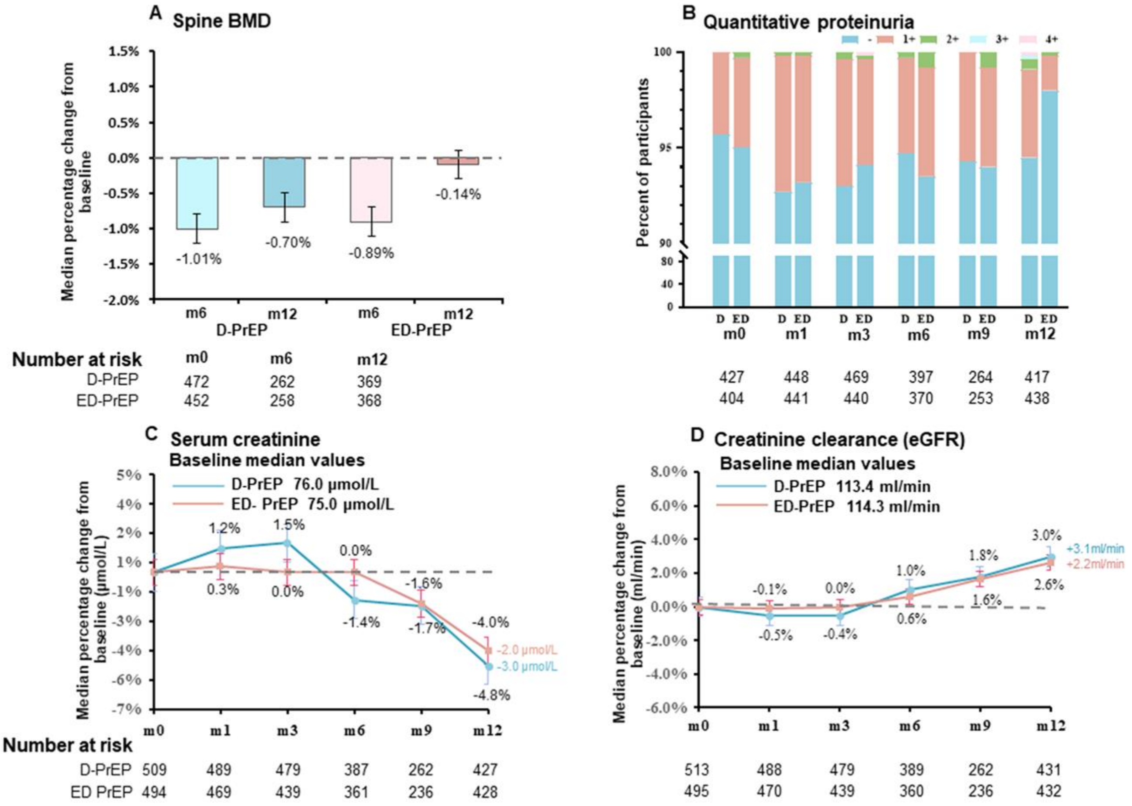

BMD= bone mineral density; D-PrEP=Daily PrEP; ED-PrEP=Event-driven PrEP.
Missing data due to COVID-19 failing to come to the clinic for testing.

© 2022 Wang H et al. *JAMA Network Open*.
