# Supplementary material for: Association of HIV Preexposure Prophylaxis Use With HIV Incidence Among Men Who Have Sex With Men in China: A Nonrandomized Controlled Trial
Source: JAMA Netw Open. 2022 Feb 16;5(2):e2148782. doi: 10.1001/jamanetworkopen.2021.48782 (PMC8851305; doi:10.1001/jamanetworkopen.2021.48782)
Supplement: Supplement 3. — Nonauthor Collaborators [file jamanetwopen-e2148782-s003.pdf]

Supplemental Online Content: Nonauthor Collaborators

*Indicates required information. Only first name, last name, and suffix will appear in PubMed.

*Group Name(s): China Real-World Oral Intake of PrEP (CROPReP) Study Team							
***First Name and Middle Initial(s)**	***Last Name**	***Suffix (eg, Jr, III)**	Academic Degrees	Institution	Location (city, state/province, country)	Role or Contribution, eg, chair, principal investigator	Group (if more than 1 Group listed in the byline) and/or Subgroup (eg, Steering Committee)
Rui	Li			The First Affiliated Hospital of China Medical University			
Qiang	Kang			The First Affiliated Hospital of China Medical University			
Shangcao	Li			The First Affiliated Hospital of China Medical University			
Zhili	Hu			The First Affiliated Hospital of China Medical University			
Rantong	Bao			The First Affiliated Hospital of China Medical University			
Hang	Li			The First Affiliated Hospital of China Medical University			
Yonghui	Zhang			The First Affiliated Hospital of China Medical University			
Zhu	Mei			The First Affiliated Hospital of China Medical University			
Yueru	Jia			The First Affiliated Hospital of China Medical University			
Zehao	Ye			The First Affiliated Hospital of China Medical University			
Yanni	Ma			The First Affiliated Hospital of China Medical University			
Xin	Ma			The First Affiliated Hospital of China Medical University			
Xiaoyun	Shi			The First Affiliated Hospital of China Medical University			
Yijun	Duan			Beijing Youan Hospital			
Guanghui	Zhang			Beijing Youan Hospital			
Fang	Zhao			Shenzhen Third People's Hospital			

Supplemental Online Content: Nonauthor Collaborators

*Indicates required information. Only first name, last name, and suffix will appear in PubMed.

***First Name and Middle Initial(s)**	***Last Name**	***Suffix (eg, Jr, III)**	Academic Degrees	Institution	Location (city, state/province, country)	Role or Contribution, eg, chair, principal investigator	Group (if more than 1 Group listed in the byline) and/or Subgroup (eg, Steering Committee)
Yao	Li			Chongqing Public Health Medical Center			
