# Supplementary material for: Association of HIV Preexposure Prophylaxis Use With HIV Incidence Among Men Who Have Sex With Men in China: A Nonrandomized Controlled Trial
Source: JAMA Netw Open. 2022 Feb 16;5(2):e2148782. doi: 10.1001/jamanetworkopen.2021.48782 (PMC8851305; doi:10.1001/jamanetworkopen.2021.48782)
Supplement: Supplement 4. — Data Sharing Statement [file jamanetwopen-e2148782-s004.pdf]

Data Sharing Statement

Wang. Association of HIV Pre-exposure Prophylaxis Use With HIV Incidence Among Men Who Have Sex With Men in China. *JAMA Netw Open*. Published February 16, 2022. doi:10.1001/jamanetworkopen.2021.48782

Data
Data available: No
